# Low knowledge of antiretroviral treatments for the prevention of HIV among precarious immigrants from sub-Saharan Africa living in the greater Paris area: Results from the Makasi project

**DOI:** 10.1371/journal.pone.0287288

**Published:** 2023-06-14

**Authors:** Karna Coulibaly, Anne Gosselin, Severine Carillon, Corinne Taéron, Romain Mbiribindi, Annabel Desgrées Du Loû, on behalf of the Makasi study group

**Affiliations:** 1 Université Paris Cité, IRD, INSERM, Ceped, Paris, France; 2 French Collaborative Institute on Migration, CNRS, Aubervilliers, France; 3 French Institute for Demographic Studies (INED), Aubervilliers, France; 4 Arcat, Paris, France; 5 Afrique Avenir, Paris, France; 6 Institute for Research on Sustainable Development (IRD), Bondy, France; Aga Khan University - Kenya, KENYA

## Abstract

**Introduction:**

In France, combination prevention tools, particularly antiretroviral treatment for HIV prevention has been available for several years. We described the knowledge of these antiretroviral treatments among immigrants from sub-Saharan Africa, who are particularly affected by HIV, and the factors associated with this knowledge.

**Methods:**

The data come from the Makasi study, which was conducted between 2019 and 2020 among precarious immigrants from sub-Saharan Africa recruited through a community-based outreach approach in the greater Paris area (n = 601). We described levels of knowledge of HIV treatment effectiveness (HTE), treatment as prevention (TasP), post-exposure prophylaxis (PEP), and pre-exposure prophylaxis (PrEP), by sex with chi2 test. We investigated factors associated with their knowledge with logistic regressions adjusted for sociodemographic characteristics, living conditions and sexual behaviors (p≤0.2).

**Results:**

Respondents were mostly men (76%), from West Africa (61%), in precarious situation: 69% were unemployed, 74% were undocumented and 46% had no health coverage. Among this population, knowledge of HIV preventive treatments was heterogeneous. While HTE was well known (84%); TasP was known by only half of the respondents (46%), and PEP and PrEP were hardly known: 6% and 5%, respectively. Multivariate regressions models showed that these antiretroviral treatments for the prevention of HIV was better known by people with a higher level of education (PEP: aOR = 3.33 [1.09–10.20], p = 0.03; HTE: aOR = 4.33 [1.87–10.04], p<0.001), those who had a social network in France (TasP: aOR = 1.90, [1.33–2.73], p<0.001), those who had access to the health system and those who were exposed to sexual risks (TasP: aOR = 3.17, [1.03–9.69], p = 0.04; PrEP: aOR = 2.60 [0.72–9.34], p = 0.14).

**Conclusions:**

There is a need for specific communication on antiretroviral treatment for HIV prevention that targets sub-Saharan immigrants, particularly those who have no access to the health-care system and those who are less educated.

## Introduction

Immigrants from sub-Saharan Africa living in Europe are particularly affected by HIV infection [1]. In France, around 37% of new HIV diagnoses in 2018 were among immigrants born in sub-Saharan Africa [2]. Studies have shown that these infections are linked not only to epidemic situations in the countries of origin but also to infection in the country of immigration. In France, it is estimated that between 35% and 49% of Africans living with HIV acquired the infection after their migration [3]. The ANRS-Parcours survey showed that social hardship encountered in France is an indirect factor of exposure to HIV infection since being without stable housing or a residence permit increases the risk of having unprotected sex, sex with casual partners and experiencing sexual violence for women [3–5].

Since several years, HIV combination prevention tools have been available to prevent new infections. These prevention tools include condoms use, HIV testing and the use of antiretroviral preventive treatments such as postexposure prophylaxis (PEP) [6], pre-exposure prophylaxis (PrEP) [7] and treatment as prevention (TasP) [8]. PEP was adopted in France as an HIV prevention tool in 1998. It is a combination of antiretroviral drugs taken by HIV-negative persons who have been exposed to the risk of HIV contamination in the professional environment, through unprotected sex or through the use of a dirty needle. It must be taken within 48 hours after exposure to the virus, then every day for up to a month [9]. PrEP, available in France since 2016 [10], refers to antiretroviral drugs taken by HIV-negative persons before potential exposure to the virus to reduce the risk of HIV infection. TasP has been described in 2008. It refers to the fact that people living with HIV who are on antiretroviral treatment and who achieve and maintain an undetectable viral load beyond six months do not transmit HIV to their sexual partners [11]. In France, it is promoted as a tool of HIV prevention since 2010 [12].

The prescription of these prevention tools, which are reimbursed by social security, is recommended by the French health authorities in populations most vulnerable to HIV acquisition, mainly men who have sex with men (MSM) and people born in sub-Saharan Africa [10]. However, to date, limited research has been conducted to assess the state of knowledge of these treatments among immigrants from sub-Saharan Africa, both in France and in other Western countries [13].

In France, available quantitative studies have focused on HIV testing and condom use in this population. The Knowledge, attitudes, beliefs and practices (KABP) survey [14] conducted in 2005 among 1874 immigrants from sub-Saharan Africa aged 18 to 49 living in the greater Paris area showed that 65% of respondents had been tested for HIV a least once in their lifetime. In 2016, another survey conducted in the same population showed similar results: 66% of respondents had been tested for HIV in the 12 months prior to the survey and 15% more than 12 months ago; condom use was 57% in the past 12 months among sexually active individuals [15]. Regarding antiretroviral preventive treatments, the studies are exploratory, mainly qualitative and focused on the dissemination and acceptance of PrEP and the difficulties associated with it, but they did not measure the level of knowledge of these prevention methods [16–18].

Using data from the Makasi survey, this study aimed to measure the level of knowledge of these prevention tools and to study the factors associated with knowledge of HIV antiretroviral preventive treatments among immigrants from sub-Saharan Africa living in precarious situations in the greater Paris area.

## Materials and methods

### Study design and participants

The data used were collected at baseline during the Makasi project, a community-based participatory research that was designed and conducted by researchers and two community-based organizations (Afrique Avenir and ARCAT) as well as a peer groups [19]. The CBO members were part of the steering committees and involved in all aspects of the project. The project aimed to reduce social vulnerability and improve sexual health empowerment among immigrants from sub-Saharan Africa to reduce the risk of HIV infection.

Participants were recruited from intervention sites of the community-based organization Afrique Avenir, which offers rapid outreach testing and health sensitization. Participants were born in sub-Saharan Africa, were 18 years of age or older, HIV-negative and met at least one of the following vulnerability criteria: no stable housing, unemployed, have experienced food deprivation in the last month, have no residence permit or short-term residence permit, are or have been victims of violence, have no health coverage, feel isolated and do not know where to go to see a doctor. Participants received 10 € vouchers for their participation. The survey was conducted in French and English. At inclusion, all participants completed questionnaires administered face-to-face by trained health mediators. These questionnaires included questions on sociodemographic characteristics, living conditions, sexual behavior, and knowledge of combination prevention tools. The study protocol is registered on Clinicaltrials.gov (NCT04468724) and a description of the project has been published [19].

### Ethical consideration

The Makasi study was approved by the French Data Protection Authority (Commission Nationale de l’Informatique et des Libertés, CNIL, declaration n°2215270) and the Committee for Persons’ Protection (Comité de Protection des Personnes, CPP, Sud-Ouest et Outre-Mer, ID RCB 2018-A02129-46). All participants provided written informed consent.

### Variables of interest

First, we studied variables related to knowledge of the health-care system and HIV prevention practices such as knowledge of sexual health services, HIV testing, ability to decide how to protect oneself against HIV, condom use at last occasional sex, and perceived risk of HIV infection (for more details, please see S1 Table). Next, the knowledge of four variables of antiretroviral treatment for the prevention of HIV was studied in detail; these included knowledge of HIV treatment effectiveness (HTE), knowledge of treatment as prevention (TasP), knowledge of pre-exposure prophylaxis (PrEP), and knowledge of post-exposure prophylaxis (PEP).

For HTE and TasP, participants were asked to say whether they completely agree/more or less agree/do not really agree/completely disagree with the following statements, “I think that thanks to treatments, a person who has HIV can have a normal life” (HIV treatment effectiveness); “I think that someone who has HIV and takes his or her medicine does not transmit HIV when having sex” (TasP). For the analyses, responses were coded “Yes” for those who responded that they completely agreed or more or less agreed and “No” for those who responded that they did not agree or completely disagreed.

Questions related to PEP and PrEP were formulated as follows: “Have you heard about post-exposure prophylaxis (or emergency treatment) that you take very quickly AFTER sex to prevent HIV transmission?” (PEP: yes/no), and “Have you heard about pre-exposure prophylaxis (PrEP), a treatment that you take BEFORE sex without condoms and that protects you from HIV?” (PrEP: yes/no).

### Independent variables

The association between the variables of interest and the independent variables was tested. The independent variables were sociodemographic characteristics: sex, age (18–29, 30–39, and 40+), educational level (none/primary, secondary, superior), region of birth (West Africa, other parts of sub-Saharan Africa), main reasons for coming to France (work or study, family, medical and other, threat), and duration of stay in France in years (0–2, 3–6, 7+). Variables related to contacts with the health system were used: health insurance coverage (no health insurance coverage; state medical assistance (SMA), i.e., social aid mainly intended to cover the medical expenses of undocumented foreigners living in France; universal health insurance coverage (UHC)), and children (yes/no). Living conditions and social situation in France were captured by occupational status (unemployed, employed (informal/formal/student)), housing situation (own housing, associations, housed by family/friends and no stable housing), residence permit (undocumented, short-term permit (less than a year) and long-term permit (1 year or more), having someone close you can rely on in times of hardship (yes/no) and empowerment in sexual health scores (4 quartiles). This score was constructed from 16 questions and was validated (Cronbach’s α = 0,71) [20]. Sexual behavior variables included having a stable partnership (yes/no); having a stable or occasional same-sex sexual partner (yes/no); having transactional sex, i.e., having unwanted sex in exchange for money, papers, or other goods (yes/no); and having forced sex (yes/no).

### Statistical analysis

First, we described participants’ sociodemographic characteristics, living conditions and social situation, contact with the health-care system, and sexual behaviors by sex. We compared levels of knowledge of the health-care system, HIV-related preventive practices, and antiretroviral treatment for the prevention of HIV by sex using proportion test and displaying 95% confidence interval. Missing data were around 5% on average for some variables of interests. We performed the analysis only on the complete cases data.

Descriptive analysis using chi2 test was performed to identify variables with a p ≤ 0.2 that was considered for bivariate and multivariate analysis. The descriptive analysis was made on participants’ sociodemographic characteristics, living conditions and social situation, contact with the health-care system, and sexual behaviors to check whether or not they know each antiretroviral treatment for the prevention of HIV (see S1 Table). Finally, we performed bivariate analysis and multivariate nested logistic regression models to investigate factors associated with knowledge of each type of treatment. Multivariate logistic regression models were adjusted for sociodemographic variables as well as all other variables with a p ≤ 0.2. Multivariate logistic regression models were nested as follows:

- Model 1: sociodemographic characteristics
- Model 2: sociodemographic characteristics + contact with the health-care system
- Model 3: sociodemographic characteristics + contact with the health-care system + social situation in France
- Full model: sociodemographic characteristics + contact with the health-care system + social situation in France + sexual behaviors

The receiver operating characteristic (ROC) curves [21] were used to perform sensitivity and specificity analysis for each of the models. The goodness-of-fit of the models was also investigated using the Hosmer and Lesmeshow goodness-of-fit test [22].The analysis were performed in Stata 15 (Stata Corporation, College Station, TX, USA).

## Results

**Fig 1:**
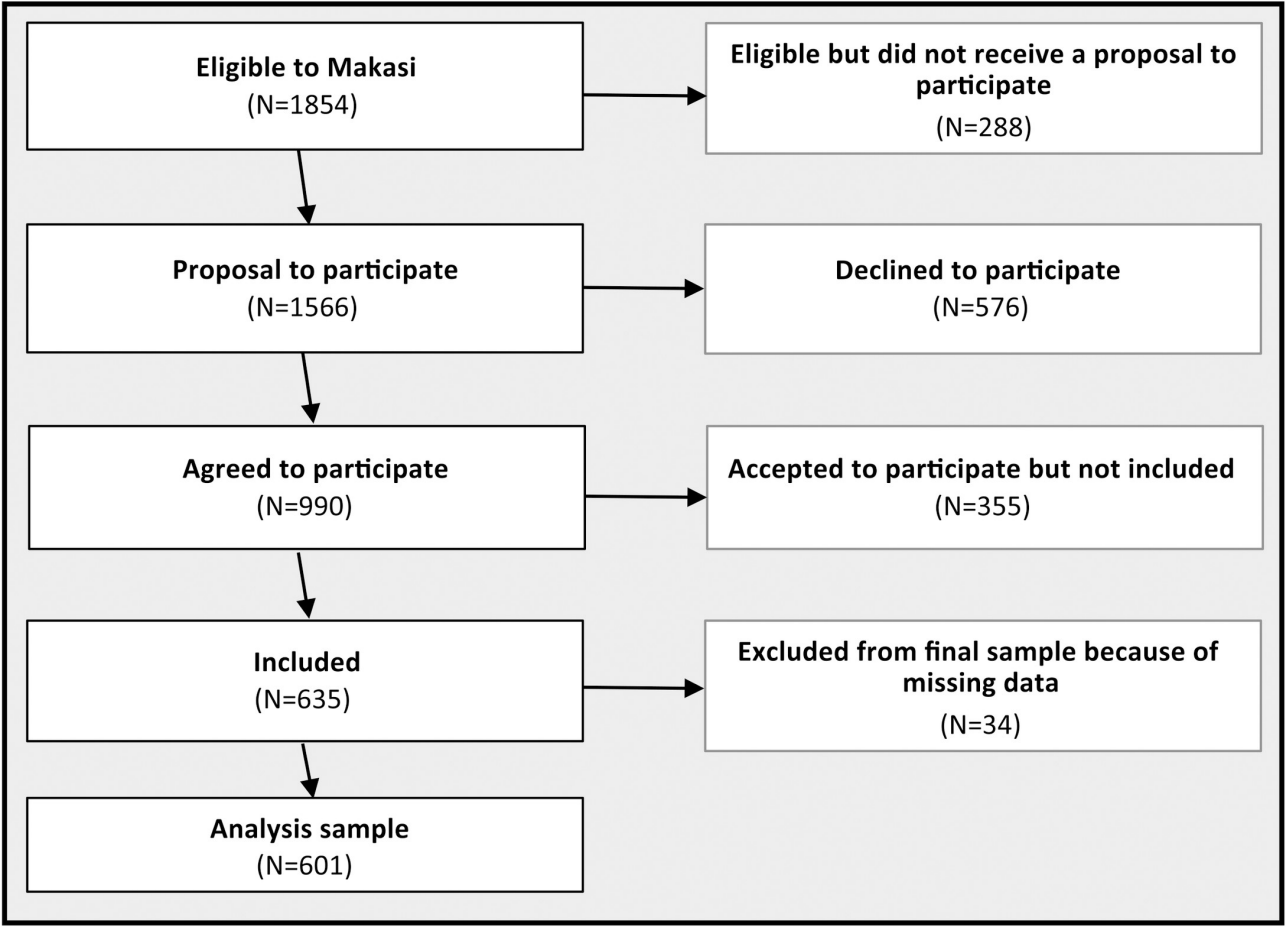
Recruitment flowchart.

Between February 2019 and December 2020, of the 1854 individuals who were eligible for participation in the Makasi project, 1566 (84%) received a proposal to participate, 990 (53%) agreed to participate and 635 (34%) participants were included (Fig 1). Some respondents were lost between acceptance and inclusion because the project took place in public spaces and on trucks. On busy days, people had to wait in a tent to be received in the inclusion truck after they accepted to participate, and some refused to wait. 34 individuals were excluded from the analyses because of several missing values. The analyses were based on a sample of 601 participants. We compared those included in the study to those eligible but not included. Those included were similar to those not included in terms of region of birth. They differed significantly on other socio-demographic characteristics such as sex, age, duration of stay in France and living conditions (see S2 Table). Those included in the study were more often men (76.2 vs 66.5; p<0,001), arrived in France in the two years before the study (50.1 vs 30.8, p<001) and they were more precarious: unemployed (69.2 vs 59.6, p<0.001); undocumented (73.9 vs 48.6, p<0.001).

### Sociodemographic characteristics and knowledge levels of combination prevention tools

A predominantly male population and many precarious situations

The characteristics of the study population are described in Table 1. It was a predominantly male population (76.2% were men), largely young (30.6% are under 30 years of age) and educated (51.2% had secondary schooling and 18.5% had superior education). Half of the participants had arrived in the two years preceding the survey (50.1%), often to work (including to study): 47.2 of men; 47.6 of women; or to flee their country: 40.8 of men; 35.7 of women.

**Table 1:** Socio-demographic characteristics, living conditions and sexual behaviours by sex.

	Men		Women		All	
	N	%	N	%	N	%
**Sociodemographic characteristics**						
	458	100%	143	100%	601	100%
**Sex**						
Men					458	76.2
Women					143	23.8
**Age (years)**						
18–29	123	26.9	61	42.7	184	30.6
30–39	208	45.4	44	30.8	252	41.9
40 +	127	27.7	38	26.6	165	27.5
**Educational level**						
None/Primary	140	30.6	42	29.4	182	30.3
Secondary	228	49.8	80	55.9	308	51.2
Superior	90	19.7	21	14.7	111	18.5
**Can talk in French**						
Fluent	368	80.3	98	68.5	466	77.5
No/can manage	90	19.7	45	31.5	135	22.5
**Knows to read and write**						
No problem	320	69.9	87	60.8	407	67.7
No/little, need help	138	30.1	56	39.2	194	32.3
**Region of birth**						
West Africa	289	63.1	76	53.1	365	60.7
Other part of sub-Saharan Africa	169	36.9	67	46.9	236	39.3
**Main reason for coming to France**						
Find work/study	216	47.2	68	47.6	284	47.3
Join a family member	34	7.4	15	10.5	49	8.2
Medical reasons and other	21	4.6	9	6.3	30	5.0
Threatened in your country	187	40.8	51	35.7	238	39.6
**Duration of stay in France (years)**						
0–2	236	51.5	65	45.5	301	50.1
3–6	152	33.2	56	39.2	208	34.6
7 +	70	15.3	22	15.4	92	15.3
**Social situation**						
**Housing situation at time of survey**						
Associations	27	5.9	28	19.6	55	9.2
Housed by family/friends	242	52.8	61	42.7	303	50.4
Own housing	120	26.2	47	32.9	167	27.8
No stable housing	69	15.1	7	4.9	76	12.6
**Occupational status at time of survey**						
Unemployed	310	67.7	106	74.1	416	69.2
Employed (informal/formal/student)	148	32.3	37	25.9	185	30.8
**Have someone close you can rely on in the times of hardship**						
No	230	50.2	72	50.3	302	50.2
Yes	228	49.8	71	49.7	299	49.8
**Resident permit at time of survey**						
Undocumented	340	74.2	104	72.7	444	73.9
Short-term permit (<1 year)	78	17.0	18	12.6	96	16.0
Long-term permit (1 year and +, including French nationality)	40	8.7	21	14.7	61	10.1
**Have children**						
No	170	37.1	49	34.3	219	36.4
Yes	288	62.9	94	65.7	382	63.6
**Health insurance coverage at time of survey**						
State Medical Assistance (SMA)	116	25.3	46	32.2	162	27.0
No Health insurance Coverage	219	47.8	58	40.6	277	46.1
Universal Health insurance Coverage (UHC)	123	26.9	39	27.3	162	27.0
**Empowerment scores**						
Low (1st quartile)	101	22.1	44	30.8	145	24.1
Intermediate low (2nd quartile)	129	28.2	41	28.7	170	28.3
Intermediate high (3rd quartile)	132	28.8	37	25.9	169	28.1
High (4th quartile)	96	21.0	21	14.7	117	19.5
**Sexual behaviors**						
**Have at least one stable partnership**						
No	283	61.8	56	39.2	339	56.4
Yes	175	38.2	87	60.8	262	43.6
**Have at least one stable or occasional same-sex sexual partnership in the last 12 months**						
No	316	69.0	121	84.6	437	72.7
Yes	22	4.8	3	2.1	25	4.2
No sexual partner	120	26.2	19	13.3	139	23.1
**Occasional partnership in the last 12 months**						
No	222	48.5	81	56.6	303	50.4
Yes	236	51.5	62	43.4	298	49.6
**Transactional sex**						
No	440	96.1	113	79.0	553	92.0
Yes	18	3.9	30	21.0	48	8.0
**Forced sex**						
No	454	99.1	131	91.6	585	97.3
Yes	4	0.9	12	8.4	16	2.7

Source: Makasi survey, 2019–2020

The survey concerns a population that often faces precarious situations. One person out of two was housed by family or friends (50.4%), and only 27.8% of the respondents had their own housing. There was also a large proportion of people who were unemployed (69.2%), undocumented (73.9%) and without health insurance coverage (46.1%).

Nearly half of the respondents had a stable primary partner (43.6%), and this was more often the case for women than for men (60.8% vs. 38.2%). Most of these partners were of the opposite sex, with only 4.2% of participants having had stable or occasional partners of the same sex in the past 12 months. 49.6% of respondents had sexual relations with occasional partners in the 12 months preceding the survey. Some respondents had transactional sex (8%) and forced sex (2.7%), with higher levels among women (21% had transactional sex and 8.4% had experienced forced sex).

#### Heterogeneous levels of knowledge of prevention tools

Knowledge of sexual health and testing practices was high in the study population. This suggests that the study population is aware of the risk of exposure to HIV (Table 2). 70% of the respondents were aware of sexual health services, and 80% had already been tested for HIV during their lifetime, with more women than men being tested (89% vs. 78%, p = 0.003). Regarding the ability to decide how to protect oneself, 81% declared that it was fairly easy or very easy to decide how to protect oneself, with men giving this answer more than women (83% vs. 75%, p = 0.03). This result appears to be consistent with protection at last occasional sexual encounter: more men reported using a condom at last occasional sexual encounter (57% vs. 47% among women). We also noted that 41% of respondents reported having a perceived risk of HIV infection equal to or more than the rest of the population.

**Table 2:** Levels of knowledge and behaviour related to HIV prevention by sex.

	Level of knowledge						
	All		Men		Women		
	n/N	% (95% CI)	n/N	% (95% CI)	n/N	% (95% CI)	p-values†
**Knowledge of sexual health services, HIV protection behaviors and risk perception**							
Knowledge of sexual health services (N = 600)	421/600	70% (67–74)	320/457	70% (66–74)	101/143	71% (63–78)	0.89
Have been tested for HIV in life (N = 571)	460/571	80% (77–83)	337/433	78% (74–82)	123/138	89% (84–94)	0.003
Ability to decide how to protect oneself against HIV (N = 601)*	486/601	81% (78–84)	379/458	83% (79–86)	107/143	75% (68–82)	0.03
Perceived risk of HIV infection (N = 574)**	237/574	41% (37–45)	177/438	40% (36–45)	60/136	44% (36–52)	0.44
Condom use at last occasional sex among those who reported occasional sex (N = 297)	164/297	55% (50–61)	135/235	57% (51–64)	29/62	47% (34–59)	0.13
**Level of knowledge of antiretroviral treatment for the prevention of HIV**							
Knowledge of HIV treatment effectiveness (HTE) (N = 601)	502/601	84% (81–86)	389/458	85% (82–88)	113/143	79% (72–86)	0.09
Knowledge of HIV treatment as prevention (TasP) (N = 601)	274/601	46%(42–50)	214/458	47% (42–51)	60/143	42%(34–50)	0.31
Knowledge of post-exposure prophylaxis (PEP) (N = 519)	32/519	6% (4–8)	24/394	6% (4–8)	8/125	6% (2–11)	0.90
Knowledge of pre-exposure prophylaxis (PrEP) (N = 519)	27/519	5% (3–7)	20/394	5% (3–7)	7/125	6% (2–10)	0.81

Source: Makasi survey, 2019–2020

† chi2 test comparing men and women.

* Very easy or rather easy to decide how to protect oneself against HIV

**Perceived risk of HIV infection same to or more than the rest of the population

Knowledge of antiretroviral treatment for the prevention of HIV was very heterogeneous (Table 2). While 84% of the respondents knew about HTE and nearly half knew about TasP (46%), only 5% and 6% knew about PrEP and PEP, respectively.

#### Factors associated with knowledge of antiretroviral treatment for the prevention of HIV

**Fig 2:**
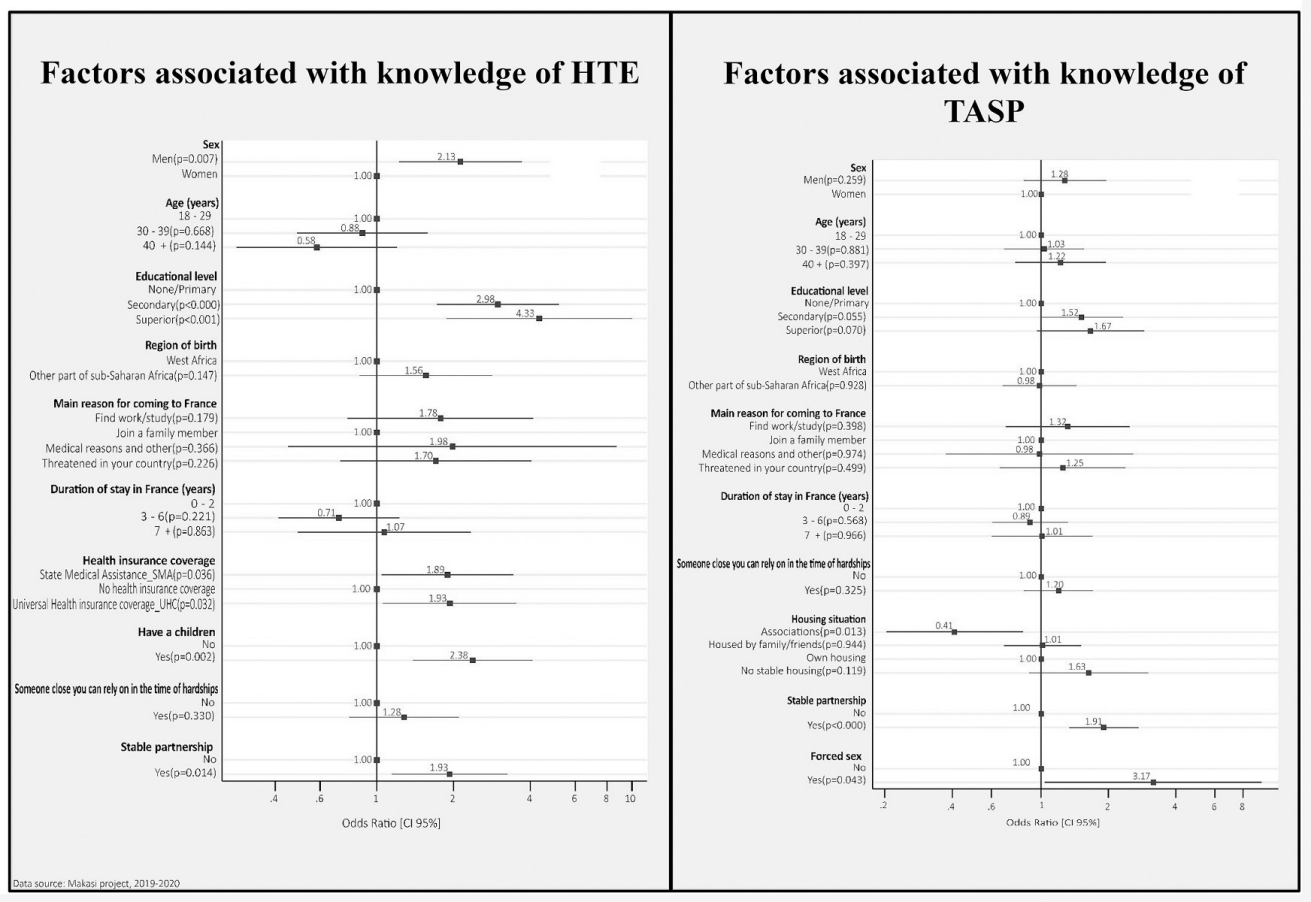
Factors associated with the knowledge of HTE and TASP.

**Fig 3:**
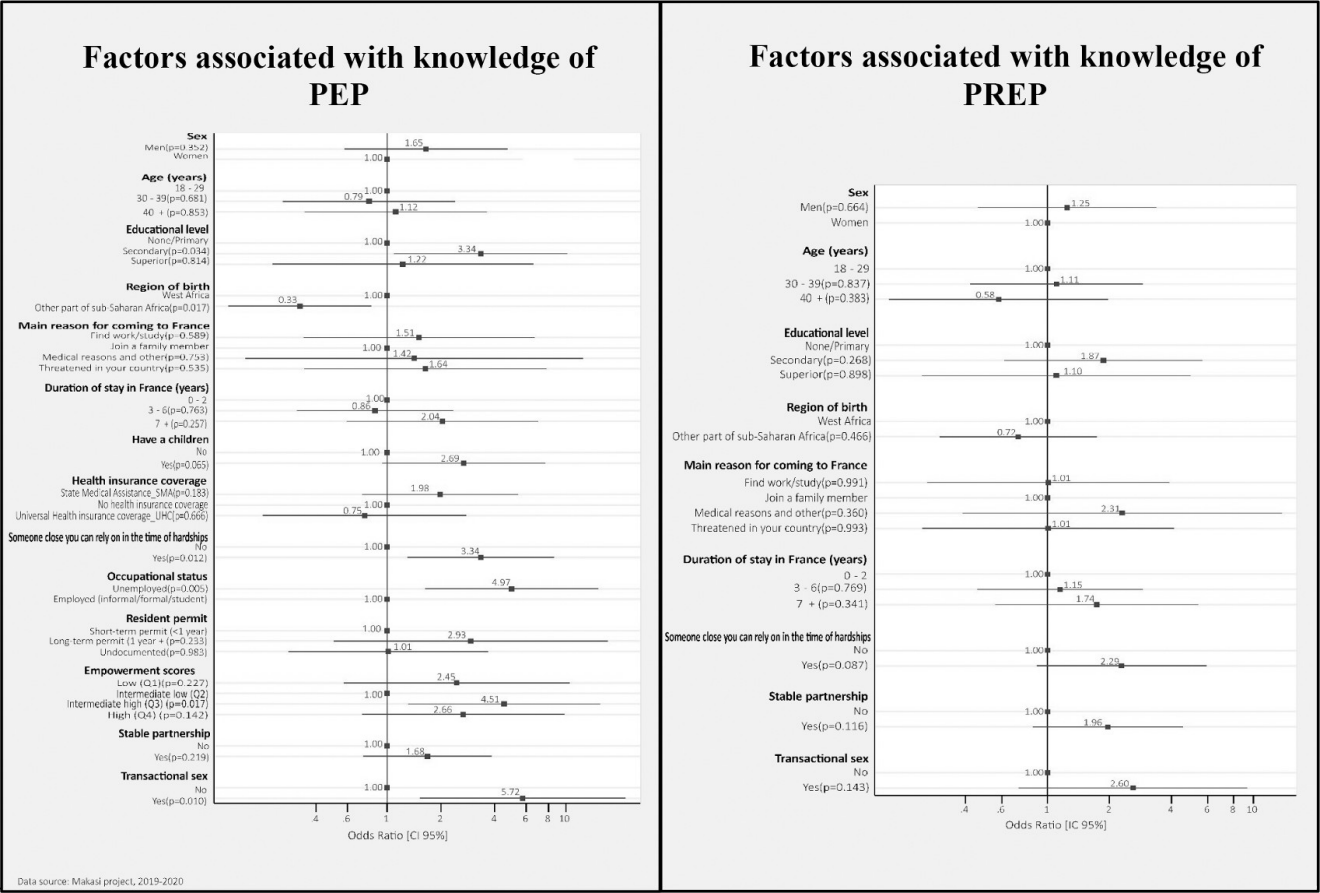
Factors associated with the knowledge of PEP and PrEP.

We presented the results of the full multivariate logistic regression models in the article (Figs 2 and 3). Results on Models 1, 2 and 3 are in the supplemental supporting information (see S3–S6 Tables). Goodness-of-fit tests and areas under the ROC curves showed that the multivariate logistic regression models had good properties (see S3–S6 Tables).

#### Factors associated with knowledge of HTE and TasP

Men were twice as likely as women to be aware of HTE (aOR = 2.12 [1.22–3.69], p = 007) (Fig 2). Those with secondary education (aOR = 2.98 [1.71–5.18], p<0.001) and superior education (aOR = 4.33 [1.87–10.04], p<0.001) were more likely to have this knowledge than those with primary education. Having children and having an SMA or UHC increased the probability of knowing about HTE (aOR = 2.37 [1.38–4.09], p = 0.002; aOR = 1.89 [1.04–3.44], p = 0.03 and aOR = 1.93 [1.05–3.53], p = 0.03, respectively). The same was true for having a stable partnership (aOR = 1.93 [1.14–3.26], p = 0.01). Having someone to rely on in France was significantly associated to knowledge of HTE in the bivariate analysis (OR = 1.73 [1.11–2.70], p = 0.01) but this association was no longer significant in the multivariate analysis.

In the multivariate model 1 adjusted for participants’ sociodemographic, we saw that those with a secondary or superior education levels were more likely to know about TasP than those with a primary education level (aOR = 1.52[1.01–2.30], p = 0.043 and aOR = 1.71[1.00–2.91, p = 0.048, respectively). This results is also confirmed in the full model, even less significant (aOR = 1.51 [0.99–2.32], p = 0.055 and aOR = 1.66 [0.95–2.89], p = 0.070, respectively) (Fig 2). Having a stable partner or having experienced forced sex also increased the probability of knowing about TasP (aOR = 1.90 [1.33–2.73], p<0.001) and aOR = 3.17 [1.03–9.69], p = 0.04, respectively).

#### Factors associated with knowledge of PEP and PrEP

Those with a secondary education were three times more likely to know about PEP than those with a primary education (aOR = 3.33 [1.09–10.20], p = 0.03) (Fig 3). This probability was also higher for those who were unemployed than for those who were employed (aOR = 4.97 [1.62–15.18], p = 0.005). In addition, having someone to rely on in France increased the probability of knowing about PEP (aOR = 3.34 [1.30–8.59], p = 0.01). People with a high intermediate level of sexual empowerment had a significantly higher probability of knowing about PEP than those with a low intermediate level of sexual empowerment (aOR = 4.51 [1.30–15.60], p = 0.01). Having transactional sex significantly increased the probability of knowing about PEP (aOR = 5.72 [1.51–21.57], p = 0.01).

In the full model on the factors associated with knowledge of PrEP, none of the variables were significant at the 5% level. However, we observe certain trends. For example, having someone to rely on in France appears to be correlated with the knowledge of PrEP (aOR = 2.28 [0.88–5.90], p = 0.08). Similarly, having a stable primary partner and experiencing transactional sex appears to be correlated with the knowledge of PrEP (aOR = 1.96 [0.84–4.54], p = 0.11 and aOR = 2.60 [0.72–9.34], p = 0.14, respectively) (Fig 3). These trends were consistent with the fact that having someone to rely on in France and having a stable primary partner were more significant in the bivariate analysis (OR = 2.57[1.06–6.18], p = 0.03 and OR = 2.22 [0.99–4.96], p = 0.050 respectively).

## Discussion

This study population faces several precarious situations. In this study, most participants were young and male, half of them had recently arrived in France. This participants are rarely surveyed in other studies [23]. Our results showed that in this population, the levels of knowledge of antiretroviral treatment for the prevention of HIV were heterogeneous: the effectiveness of HIV treatment was widely known, half of the respondents knew about TasP, while less than 10% of them were aware of PrEP and PEP.

We can see from our results three categories of factors that were linked to the knowledge of HIV preventive treatments: access to information, contact with the health-care system, and exposure to sexual risks.

The participants who were informed about antiretroviral treatment for the prevention of HIV were those who had access to information in general: the people with a higher level of education and those who knew someone in France (i.e., they had someone to rely on; this person could also be a partner). As highlighted in work on PrEP in the United States, this information on antiretroviral treatment for the prevention of HIV is generally shared on social media and in interpersonal relationships [24]. Therefore, in France, we found that personal networks and good health literacy play an important role in the exposure to information.

Participants who had UHC or SMA were more likely to have access to the health-care system than others; this is also the case for people who had a child. As expected, this proximity to the health system is associated to the knowledge of antiretroviral treatment for the prevention of HIV.

Finally, people who were exposed to sexual risks (those who reported transactional relationships or those who experienced forced sex) were more aware of antiretroviral treatment for the prevention of HIV, probably because they were more targeted by information campaigns on these treatments, as suggested in the HIV prevention strategies in France [25]. Compared to persons who have no stable partner, people who have a stable partner are also more likely to be more aware of antiretroviral treatment for prevention of HIV. These results suggest that being sexually active is a factor positively correlated with knowledge of antiretroviral treatment for the prevention of HIV.

The results of this research reveal a low levels of knowledge of the main preventive treatments for HIV-negative African immigrant living in precarious situation in France. This result is similar to what has been shown in the United Kingdom, where research has also highlighted low levels of knowledge of PrEP and PEP in a population composed mainly of people from Sub-Saharan Africa: Only 19% of respondents had heard of PrEP, and 59% knew about PEP [26]. A recent study in Portugal reported 4,5% of PEP knowledge and 2,3% of PrEP knowledge among immigrant born in Africa [27]. In France, at the time of this study, although there has been no other survey carried out among immigrants from sub-Saharan Africa with which to compare our results, the number of people who were aware of PrEP and PEP in our study was low compared to the data available among MSM [28–31]. Indeed, the EMIS survey reported that among MSM surveyed in France, 59% were aware of TasP in 2017. Knowledge of PrEP and PEP were 83% and 69%, respectively [30]. In the PREVAGAY study conducted in late 2015, 77% knew about PEP, and 54% knew about PrEP [31]. Despite the differences and possible links between African immigrant, particularly those in precarious situations, and MSM, our results point out an important knowledge gap between this two populations exposed to HIV, as also reported in Portugal [27].

This knowledge gap may be linked to a lack of communication strategies targeting precarious immigrants, as recommended by French health authorities [25]. The information campaigns initially carried out for the implementation of clinical trials and the populations reached by these trials were essentially urban MSM and non-immigrant population [7, 16]. Thus, the conditions under which PrEP emerged and the dissemination of information about this tool contributed to reproducing pre-existing social inequalities in health, class and race [16, 32]. This is consistent with the results from a study conducted in the United States that highlighted a lack of communication about PrEP among nonwhite populations and among non-MSM populations [24]. In France, it is recently that information campaigns on antiretroviral treatments for HIV prevention have been implemented for immigrant populations from sub-Saharan Africa [33–35]. However, it is still early to observe the effects of these campaigns on the level of knowledge of these treatments among precarious immigrants from sub-Saharan Africa.

Our study has some limitations. The study was based on a study sample of 601 people. As a result, low numbers in some of the categories did not allow for stratified analyses, such as analysis stratified by sex. In addition, the low levels of knowledge of PrEP and PEP, although they produced relevant results, impacted the statistical power of the analysis of factors associated with knowledge of these two treatments. Thus, despite the use of tests adapted to small numbers, none of the factors appeared significant at the 5% level for PrEP in the full multivariate model (n = 27). As every survey with face-to-face questionnaire, our survey may suffer from social desirability bias. However, sexual indicators (eg same sex relationships) are similar to those obtained in other surveys in this population [36]. Desirability bias does not seem to impact our results in terms of levels of knowledge of antiretroviral treatments for HIV prevention insofar as we find heterogeneous levels of knowledge according to the indicators and that similar results have been observed in the few existing studies. People included in the study were arrived more recently and lived in more difficult socio-economic situations that people eligible but not included. This bias may amplify the poor knowledge of antiretroviral prevention. Furthermore, concerning the variable “having a stable partnership”, given that no distinction was made between a stable partnership in France or in the country of origin, it is possible that some respondents declared stable relationships with partners who do not live in France. It is also possible that the stability of the partner was not understood in the same way by the respondents. Finally, the results presented here cannot be generalized to the entire immigrant population of Sub-Saharan African origin residing in the greater Paris area, since our study population is selected on the basis of precarious situations. Despite these limitations, this study has the merit of being one of the few to describe levels of knowledge of HIV preventive treatments in an immigrant population living in the greater Paris area and to study the factors associated with this knowledge.

## Conclusions

Our results show that there is a poor knowledge of antiretroviral treatment for the prevention of HIV among a population of precarious sub-Saharan African immigrants. Approximately one person in twenty knew PrEP and PEP, even though this population is exposed to sexual risk and the risk of HIV infection. These low levels of knowledge of PrEP and PEP suggest that prevention campaigns must be more inclusive and careful not to increase inequalities in access to information among groups exposed to HIV infection. These results highlight the need for specific communication on HIV preventive treatment for sub-Saharan immigrants in precarious situations, particularly for those who have no access to the health-care system and those with low levels of education.

Lead author for this group: Annabel Desgrées du Loû, annabel.desgrees@ird.fr

## The MAKASI Study Group member’s affiliations

^1^ Université Paris Cité, IRD, INSERM, Ceped, F-75006 Paris, France

^2^ French Collaborative Institute on Migration, CNRS, Aubervilliers, France

^3^ Institute for Research on Sustainable Development (IRD), Bondy, France

^4^ Arcat, Paris, France

^5^ IRD, UMR LEDa-DIAL, PSL, Université Paris-Dauphine, CNRS, Paris, France

^6^ Afrique Avenir, Paris, France

^7^ ERES, Social Epidemiology Unit, IPLESP, INSERM S1136, Faculté de Médecine de Saint Antoine, Paris, France

^8^ French Institute for Demographic Studies (INED), Aubervilliers, France

^9^ Vers Paris sans Sida, Paris, France

^10^ RITM, Université Paris-Saclay, Sceaux, France

## Supporting information

S1 Checklist The STROBE Statement checklist. (PDF)

S1 Text Indicators and items used in this article. (PDF)

S1 Table Sociodemographic characteristics, living conditions and sexual behaviours by status of knowledge of antiretroviral treatment for the prevention of HIV, by sex. (PDF)

S2 Table Sociodemographic characteristics of those eligible for Makasi, by whether or not they were included in the study. (PDF)

S3 Table Factors associated with knowledge of HIV treatment effectiveness (HTE). Nested logistic regression models.
(PDF)

S4 Table Factors associated with knowledge of treatment as prevention (TasP). Nested logistic regression models.
(PDF)

S5 Table Factors associated with knowledge of post-exposure prophylaxis (PEP). Nested logistic regression models.
(PDF)

S6 Table Factors associated with knowledge of pre-exposure prophylaxis (PrEP). Nested logistic regression models.
(PDF)
