# Supplementary material for: Low knowledge of antiretroviral treatments for the prevention of HIV among precarious immigrants from sub-Saharan Africa living in the greater Paris area: Results from the Makasi project
Source: PLoS One. 2023 Jun 14;18(6):e0287288. doi: 10.1371/journal.pone.0287288 (PMC10266671; doi:10.1371/journal.pone.0287288)
Supplement: S1 Checklist — (PDF) [file pone.0287288.s001.pdf]

Appendix 8: The STROBE Statement—Checklist of items that should be included in reports of cross-sectional studies applied to the Makasi Study

	Item N°	Recommendation	Pages
**Title and abstract**	1	***(a)*** **Indicate the study's design with a commonly used term in the title or the abstract**<br>See abstract	Manuscript p.3-4
		(b) **Provide in the abstract an informative and balanced summary of what was done and what was found**<br>See abstract	Manuscript p.3-4
**Introduction**			
Background/rationale	2	**Explain the scientific background and rationale for the investigation being reported**<br>See Introduction	Manuscript p.5-6
Objectives	3	**State specific objectives, including any prespecified hypotheses:**<br>See Introduction	Manuscript p.5-6
**Methods**			
Study design	4	**Present key elements of study design early in the paper:**<br>See study design and participants in Material and Methods section	Manuscript p.7
Setting	5	**Describe the setting, locations, and relevant dates, including periods of recruitment, exposure, follow-up, and data collection**<br>See study design and participants in Material and Methods section	Manuscript p.7
Participants	6	***(*a) Give the eligibility criteria, and the sources and methods of selection of participants**<br>See study design and participants in Material and Methods section	Manuscript p.7
Variables	7	**Clearly define all outcomes, exposures, predictors, potential confounders, and effect modifiers. Give diagnostic criteria, if applicable.**<br>See variables of interest and Independent variables in Material and Methods section	Manuscript p.8-9
Data sources/measurement	8*	**For each variable of interest, give sources of data and details of methods of assessment (measurement). Describe comparability of assessment methods if there is more than one group**<br>See variables of interest and Independent variables in Material and Methods section	Manuscript p.7
Bias	9	**Describe any efforts to address potential sources of bias:**<br>See study design and participants in Material and Methods section	Manuscript p.8-9
Study size	10	**Explain how the study size was arrived at:**	

		See results section	Manuscript p.11
Quantitative variables	11	**Explain how quantitative variables were handled in the analyses. If applicable, describe which groupings were chosen and why.** See statistical analysis in Material and Methods section	Manuscript p.9-10
Statistical methods	12	***(*a) Describe all statistical methods, including those used to control for confounding** See statistical analysis in Material and Methods section	Manuscript p.9-10
		***(*b) Describe any methods used to examine subgroups and interactions** See statistical analysis in Material and Methods section	Manuscript p.9-10
		***(*c) Explain how missing data were addressed** See statistical analysis in Material and Methods section	Manuscript p.9-10
		**(d) If applicable, describe analytical methods taking account of sampling strategy** Not applicable	
		**(*e*) Describe any sensitivity analysis** See statistical analysis in Material and Methods section	Manuscript p.9-10
**Results**			
Participants	13*	**(a) Report numbers of individuals at each stage of study—eg numbers potentially eligible, examined for eligibility, confirmed eligible, included in the study, completing follow-up, and analysed** See results section	Manuscript p.11
		**(b) Give reasons for non-participation at each stage** See results section	Manuscript p.11
		**(c) Consider use of a flow diagram** See results section	Manuscript p.11
Descriptive data	14*	**(a) Give characteristics of study participants (eg demographic, clinical, social) and information on exposures and potential confounders** See results section	Manuscript p.11-12
		**(b) Indicate number of participants with missing data for each variable of interest** See results section	Manuscript p.14-15
Outcome data	15*	**Report numbers of outcome events or summary measures** See results section	Manuscript p.14-15
Main results	16	**(a) Give unadjusted estimates and, if applicable, confounder-adjusted estimates and their precision (eg, 95% confidence interval). Make clear which confounders were adjusted for and why they were included** See results section and supporting information (S4-S7 Tables)	Manuscript p.16-17
		**(b) Report category boundaries when continuous variables were categorized** See results section	Manuscript p.13-15
		*(*c) If relevant, consider translating estimates of relative risk into absolute risk for a meaningful time period ***Not applicable***	
Other analyses	17	**Report other analyses done—eg analyses of subgroups and interactions, and sensitivity analyses**	

		See supporting information (S2 Table)	
**Discussion**			
Key results	18	**Summarise key results with reference to study objectives**<br>See discussion	Manuscript p.17-21
Limitations	19	**Discuss limitations of the study, taking into account sources of potential bias or imprecision. Discuss both direction and magnitude of any potential bias**<br>See discussion	Manuscript p.17-21
Interpretation	20	**Give a cautious overall interpretation of results considering objectives, limitations, multiplicity of analyses, results from similar studies, and other relevant evidence**<br>See conclusion	Manuscript p.21
Generalisability	21	Discuss the generalisability (external validity) of the study results<br>See discussion	Manuscript p.17-21
**Other information**			
Funding	22	**Give the source of funding and the role of the funders for the present study and, if applicable, for the original study on which the present article is based**<br>See Title page	Manuscript p.1
