# Supplementary material for: Low knowledge of antiretroviral treatments for the prevention of HIV among precarious immigrants from sub-Saharan Africa living in the greater Paris area: Results from the Makasi project
Source: PLoS One. 2023 Jun 14;18(6):e0287288. doi: 10.1371/journal.pone.0287288 (PMC10266671; doi:10.1371/journal.pone.0287288)
Supplement: S1 Text — (PDF) [file pone.0287288.s002.pdf]

S 1: Indicators and items used in this article

N°	Indicators	Items	Reponses modalities	Coding of responses
**1**	Knowledge of sexual health services	If you have questions about Sexually Transmitted Infections, the difficulty to have children, sexual performance (sexual problems), do you know where to go?	Yes<br>No	Yes<br>No
**2**	Have been tested for HIV in life	Have you ever been tested for HIV in your life?	Yes<br>No<br>Don't know	Yes<br>No + Don't know= No
**3**	Ability to decide how to protect oneself against HIV	Can you tell us for each of these tasks, to what extent it was easy, Rather easy, Rather difficult or difficult for you in your life in France to make a decision about how to protect oneself against HIV and STIs with the information on the radio, TV, posters, internet…?	Very easy<br>Rather easy<br>Rather difficult<br>Very difficult	Very easy or Rather easy<br>Rather difficult or Very difficult
**4**	Condom use at last occasional sex	The last time you had sex with your casual partner, did you use a condom?	Yes<br>Yes, but the condom breaks<br>No	Yes + Yes, but the condom breaks=Yes<br>No
**5**	Perceived risk of HIV infection	Compared to others [all the population], do you think you have More risk, Same risk, Less risk, No risk at all…to be exposed to HIV virus ?	More risk<br>Same risk<br>Less risk	More risk or Same risk

			No risk at all	Less risk or No risk at all
**6**	HIV treatment effectiveness	For the following statement, can you tell me whether you completely agree/more or less agree/not really agree/completely disagree “I think that thanks to treatments, a person who has HIV can have a normal life”?	Completely agree<br>More or less agree<br>Not really agree<br>Completely disagree	Completely agree + More or less agree= Yes<br>Not really agree + Completely disagree=No
**7**	Knowledge of HIV treatment as prevention (TASP)	For each of the following statement, can you tell me whether you completely agree/more or less agree/not really agree/completely disagree “ I think that someone who has HIV and takes his/her medicines well does not transmit HIV when having sex (TASP)"?	Completely agree<br>More or less agree<br>Not really agree<br>Completely disagree	Completely agree + More or less agree= Yes<br>Not really agree + Completely disagree=No
**8**	Knowledge of pre-exposure prophylaxis (PREP)	Have you heard about the Pre-Exposure Prophylaxis (PrEP), a treatment that you take BEFORE sex without condom and that protects you from HIV?	Yes<br>No	Yes<br>No
**9**	Knowledge of post-exposure prophylaxis (PEP)	Have you heard about Post-Exposure Prophylaxis (or emergency treatment) that you take very quickly AFTER sex to prevent HIV transmission?	Yes<br>No	Yes<br>No
