# Supplementary material for: Low knowledge of antiretroviral treatments for the prevention of HIV among precarious immigrants from sub-Saharan Africa living in the greater Paris area: Results from the Makasi project
Source: PLoS One. 2023 Jun 14;18(6):e0287288. doi: 10.1371/journal.pone.0287288 (PMC10266671; doi:10.1371/journal.pone.0287288)
Supplement: S1 Table — (PDF) [file pone.0287288.s003.pdf]

S 2: *Socio-demographic characteristics, living conditions and sexual behaviours by status of knowledge of antiretroviral treatment for the prevention of HIV, by sex*

Knowledge of HIV treatment effectiveness

	**HIV treatment effectiveness Men _ (N=458)**					**HIV treatment effectiveness Women _ (N=143)**					**HIV treatment effectiveness All _ (N=601)**				
	Non		Oui		p	Non		Oui		p	Non		Oui		p
	N	%	N	%		N	%	N	%		N	%	N	%	
**Sex**															
Men											69	15.1	389	84.9	**0.09**
Women											30	21.0	113	79.0	
**Age (years)**															
18 – 29	20	16.3	103	83.7	0.80	14	23.0	47	77.0	0.60	34	18.5	150	81.5	0.67
30 – 39	32	15.4	176	84.6		7	15.9	37	84.1		39	15.5	213	84.5	
40 +	17	13.4	110	86.6		9	23.7	29	76.3		26	15.8	139	84.2	
**Educational level**															
None/Primary	41	29.3	99	70.7	**0.000**	12	28.6	30	71.4	**0.09**	53	29.1	129	70.9	**0.000**
Secondary	20	8.8	208	91.2		17	21.3	63	78.8		37	12.0	271	88.0	
Superior	8	8.9	82	91.1		1	4.8	20	95.2		9	8.1	102	91.9	
**Region of birth**															
West Africa	57	19.7	232	80.3	**0.000**	20	26.3	56	73.7	**0.09**	77	21.1	288	78.9	**0.000**
Other part of sub-Saharan Africa	12	7.1	157	92.9		10	14.9	57	85.1		22	9.3	214	90.7	
**Main reason for coming to France**															
Find work/study	37	17.1	179	82.9	0.56	13	19.1	55	80.9	0.32	50	17.6	234	82.4	0.56
Join a family member	6	17.6	28	82.4		4	26.7	11	73.3		10	20.4	39	79.6	
Medical reasons and other	3	14.3	18	85.7		0	0.0	9	100.0		3	10.0	27	90.0	
Threatened in your country	23	12.3	164	87.7		13	25.5	38	74.5		36	15.1	202	84.9	
**Duration of stay in France (years)**															

0 – 2	42	17.8	194	82.2	0.23	12	18.5	53	81.5	0.34	54	17.9	247	82.1	0.51
3 – 6	18	11.8	134	88.2		15	26.8	41	73.2		33	15.9	175	84.1	
7 +	9	12.9	61	87.1		3	13.6	19	86.4		12	13.0	80	87.0	
**Housing situation at time of survey**															
Associations	6	22.2	21	77.8	0.72	8	28.6	20	71.4	0.65	14	25.5	41	74.5	0.28
Housed by family/friends	34	14.0	208	86.0		11	18.0	50	82.0		45	14.9	258	85.1	
Own housing	18	15.0	102	85.0		9	19.1	38	80.9		27	16.2	140	83.8	
No stable housing	11	15.9	58	84.1		2	28.6	5	71.4		13	17.1	63	82.9	
**Occupational status at time of survey**															
Unemployed	48	15.5	262	84.5	0.71	21	19.8	85	80.2	0.56	69	16.6	347	83.4	0.91
Employed (informal/formal/student)	21	14.2	127	85.8		9	24.3	28	75.7		30	16.2	155	83.8	
**Have someone close you can rely on in the times of hardship**															
No	39	17.0	191	83.0	0.25	22	30.6	50	69.4	**0.005**	61	20.2	241	79.8	**0.01**
Yes	30	13.2	198	86.8		8	11.3	63	88.7		38	12.7	261	87.3	
**Resident permit at time of survey**															
Undocumented	55	16.2	285	83.8	0.41	21	20.2	83	79.8	0.74	76	17.1	368	82.9	0.69
Short-term permit (<1 year)	8	10.3	70	89.7		5	27.8	13	72.2		13	13.5	83	86.5	
Long-term permit (1 year and +, including French nationality)	6	15.0	34	85.0		4	19.0	17	81.0		10	16.4	51	83.6	
**Have children**															
No	38	22.4	132	77.6	**0.001**	11	22.4	38	77.6	0.75	49	22.4	170	77.6	**0.003**
Yes	31	10.8	257	89.2		19	20.2	75	79.8		50	13.1	332	86.9	
**Health insurance coverage at time of survey**															
State Medical Assistance (SMA)	13	11.2	103	88.8	**0.01**	8	17.4	38	82.6	0.70	21	13.0	141	87.0	**0.02**
No Health insurance Coverage	44	20.1	175	79.9		14	24.1	44	75.9		58	20.9	219	79.1	
Universal Health insurance Coverage (UHC)	12	9.8	111	90.2		8	20.5	31	79.5		20	12.3	142	87.7	

**Have at least one stable partnership**															
No	51	18.0	232	82.0	**0.02**	18	32.1	38	67.9	**0.009**	69	20.4	270	79.6	**0.004**
Yes	18	10.3	157	89.7		12	13.8	75	86.2		30	11.5	232	88.5	
**Have at least one stable or occasional same-sex sexual partnership in the last 12 months**															
No	41	13.0	275	87.0	**0.12**	26	21.5	95	78.5	0.73	67	15.3	370	84.7	0.41
Yes	3	13.6	19	86.4		1	33.3	2	66.7		4	16.0	21	84.0	
No sexual partner	25	20.8	95	79.2		3	15.8	16	84.2		28	20.1	111	79.9	
**Occasional partnership in the last 12 months**															
No	39	17.6	183	82.4	**0.14**	12	14.8	69	85.2	**0.03**	51	16.8	252	83.2	0.81
Yes	30	12.7	206	87.3		18	29.0	44	71.0		48	16.1	250	83.9	
**Transactional sex**															
No	68	15.5	372	84.5	0.25	21	18.6	92	81.4	0.17	89	16.1	464	83.9	0.39
Yes	1	5.6	17	94.4		9	30.0	21	70.0		10	20.8	38	79.2	
**Forced sex**															
No	69	15.2	385	84.8	0.39	27	20.6	104	79.4	0.72	96	16.4	489	83.6	0.80
Yes	0	0.0	4	100.0		3	25.0	9	75.0		3	18.8	13	81.3	

Knowledge of TasP

	**TasP _ Men (N=458)**					**TasP _ Women (N=143)**					**TasP _ All (N=601)**				
	Non		Oui		p	Non		Oui		p	Non		Oui		p
	N	%	N	%		N	%	N	%		N	%	N	%	
**Sex**															
Men											244	53.3	214	46.7	0.31
Women											83	58.0	60	41.9	
**Age (years)**															

18 – 29	65	52.8	58	47.2	0.68	39	63.9	22	36.1	0.45	104	56.5	80	43.5	0.63
30 – 39	115	55.3	93	44.7		23	52.3	21	47.7		138	54.8	114	45.2	
40 +	64	50.4	63	49.6		21	55.3	17	44.7		85	51.5	80	48.5	
**Educational level**															
None/Primary	84	60.0	56	40.0	**0.09**	28	66.7	14	33.3	**0.17**	112	61.5	70	38.5	**0.06**
Secondary	119	52.2	109	47.8		41	51.2	39	48.8		160	51.9	148	48.1	
Superior	41	45.6	49	54.4		14	66.7	7	33.3		55	49.5	56	50.5	
**Region of birth**															
West Africa	159	55.0	130	45.0	0.32	46	60.5	30	39.5	0.52	205	56.2	160	43.8	0.28
Other part of sub-Saharan Africa	85	50.3	84	49.7		37	55.2	30	44.8		122	51.7	114	48.3	
**Main reason for coming to France**															
Find work/study	120	55.6	96	44.4	0.82	36	52.9	32	47.1	0.68	156	54.9	128	45.1	0.97
Join a family member	18	52.9	16	47.1		9	60.0	6	40.0		27	55.1	22	44.9	
Medical reasons and other	11	52.4	10	47.6		6	66.7	3	33.3		17	56.7	13	43.3	
Threatened in your country	95	50.8	92	49.2		32	62.7	19	37.3		127	53.4	111	46.6	
**Duration of stay in France (years)**															
0 – 2	128	54.2	108	45.8	0.90	37	56.9	28	43.1	0.85	165	54.8	136	45.2	0.89
3 – 6	80	52.6	72	47.4		34	60.7	22	39.3		114	54.8	94	45.2	
7 +	36	51.4	34	48.6		12	54.5	10	45.5		48	52.2	44	47.8	
**Housing situation at time of survey**															
Associations	18	66.7	9	33.3	0.23	22	78.6	6	21.4	**0.05**	40	72.7	15	27.3	**0.02**
Housed by family/friends	133	55.0	109	45.0		30	49.2	31	50.8		163	53.8	140	46.2	
Own housing	62	51.7	58	48.3		26	55.3	21	44.7		88	52.7	79	47.3	
No stable housing	31	44.9	38	55.1		5	71.4	2	28.6		36	47.4	40	52.6	
**Occupational status at time of survey**															
Unemployed	170	54.8	140	45.2	0.33	63	59.4	43	40.6	0.56	233	56.0	183	44.0	0.23
Employed (informal/formal/student)	74	50.0	74	50.0		20	54.1	17	45.9		94	50.8	91	49.2	
**Have someone close you can rely on in the times of hardship**															

No	129	56.1	101	43.9	0.22	45	62.5	27	37.5	0.27	174	57.6	128	42.4	**0.11**
Yes	115	50.4	113	49.6		38	53.5	33	46.5		153	51.2	146	48.8	
**Resident permit at time of survey**															
Undocumented	185	54.4	155	45.6	0.52	59	56.7	45	43.3	0.39	244	55.0	200	45.0	0.51
Short-term permit (<1 year)	41	52.6	37	47.4		13	72.2	5	27.8		54	56.3	42	43.8	
Long-term permit (1 year and +, including French nationality)	18	45.0	22	55.0		11	52.4	10	47.6		29	47.5	32	52.5	
**Have children**															
No	101	59.4	69	40.6	**0.04**	23	46.9	26	53.1	**0.05**	124	56.6	95	43.4	0.41
Yes	143	49.7	145	50.3		60	63.8	34	36.2		203	53.1	179	46.9	
**Health insurance coverage at time of survey**															
State Medical Assistance (SMA)	65	56.0	51	44.0	0.28	24	52.2	22	47.8	0.32	89	54.9	73	45.1	0.21
No Health insurance Coverage	121	55.3	98	44.7		38	65.5	20	34.5		159	57.4	118	42.6	
Universal Health insurance Coverage (UHC)	58	47.2	65	52.8		21	53.8	18	46.2		79	48.8	83	51.2	
**Have at least one stable partnership**															
No	170	60.1	113	39.9	**0.000**	33	58.9	23	41.1	0.86	203	59.9	136	40.1	**0.002**
Yes	74	42.3	101	57.7		50	57.5	37	42.5		124	47.3	138	52.7	
**Have at least one stable or occasional same-sex sexual partnership in the last 12 months**															
No	158	50.0	158	50.0	**0.10**	74	61.2	47	38.8	0.20	232	53.1	205	46.9	0.55
Yes	14	63.6	8	36.4		1	33.3	2	66.7		15	60.0	10	40.0	
No sexual partner	72	60.0	48	40.0		8	42.1	11	57.9		80	57.6	59	42.4	
**Occasional partnership in the last 12 months**															
No	119	53.6	103	46.4	0.89	45	55.6	36	44.4	0.49	164	54.1	139	45.9	0.88
Yes	125	53.0	111	47.0		38	61.3	24	38.7		163	54.7	135	45.3	
**Transactional sex**															
No	239	54.3	201	45.7	**0.02**	64	56.6	49	43.4	0.50	303	54.8	250	45.2	0.52

Yes	5	27.8	13	72.2		19	63.3	11	36.7		24	50.0	24	50.0	
**Forced sex**															
No	244	53.7	210	46.3	**0.03**	77	58.8	54	41.2	0.55	321	54.9	264	45.1	**0.16**
Yes	0	0.0	4	100.0		6	50.0	6	50.0		6	37.5	10	62.5	

Knowledge of PEP

	**PEP Men _ (N=394)**					**PEP Women _ (N=125)**					**PEP All _ (N=519)**				
	Non		Oui		p	Non		Oui		p	Non		Oui		p
	N	%	N	%		N	%	N	%		N	%	N	%	
**Sex**															
Men											370	93.9	24	6.1	0.90
Women											117	93.6	8	6.4	
**Age (years)**															
18 – 29	97	95.1	5	4.9	0.34	47	92.2	4	7.8	0.43	144	94.1	9	5.9	0.23
30 – 39	170	95.0	9	5.0		40	97.6	1	2.4		210	95.5	10	4.5	
40 +	103	91.2	10	8.8		30	90.9	3	9.1		133	91.1	13	8.9	
**Educational level**															
None/Primary	117	97.5	3	2.5	**0.06**	38	95.0	2	5.0	0.28	155	96.9	5	3.1	**0.02**
Secondary	189	91.3	18	8.7		58	90.6	6	9.4		247	91.1	24	8.9	
Superior	64	95.5	3	4.5		21	100.0	0	0.0		85	96.6	3	3.4	
**Region of birth**															
West Africa	234	93.6	16	6.4	0.73	61	91.0	6	9.0	0.21	295	93.1	22	6.9	0.35
Other part of sub-Saharan Africa	136	94.4	8	5.6		56	96.6	2	3.4		192	95.0	10	5.0	
**Main reason for coming to France**															
Find work/study	178	95.2	9	4.8	0.71	53	89.8	6	10.2	0.25	231	93.9	15	6.1	0.97

Join a family member	29	90.6	3	9.4		13	100.0	0	0.0		42	93.3	3	6.7	
Medical reasons and other	16	94.1	1	5.9		7	87.5	1	12.5		23	92.0	2	8.0	
Threatened in your country	147	93.0	11	7.0		44	97.8	1	2.2		191	94.1	12	5.9	
**Duration of stay in France (years)**															
0 – 2	188	94.9	10	5.1	**0.13**	52	94.5	3	5.5	0.80	240	94.9	13	5.1	**0.12**
3 – 6	130	94.9	7	5.1		46	93.9	3	6.1		176	94.6	10	5.4	
7 +	52	88.1	7	11.9		19	90.5	2	9.5		71	88.8	9	11.3	
**Housing situation at time of survey**															
Associations	19	90.5	2	9.5	0 .79	24	100.0	0	0.0	0.23	43	95.6	2	4.4	0.69
Housed by family/friends	200	93.5	14	6.5		50	94.3	3	5.7		250	93.6	17	6.4	
Own housing	100	94.3	6	5.7		37	88.1	5	11.9		137	92.6	11	7.4	
No stable housing	51	96.2	2	3.8		6	100.0	0	0.0		57	96.6	2	3.4	
**Occupational status at time of survey**															
Unemployed	242	92.7	19	7.3	**0.16**	81	91.0	8	9.0	**0.06**	323	92.3	27	7.7	**0.03**
Employed (informal/formal/student)	128	96.2	5	3.8		36	100.0	0	0.0		164	97.0	5	3.0	
**Have someone close you can rely on in the times of hardship**															
No	174	97.2	5	2.8	**0.01**	58	95.1	3	4.9	0.50	232	96.7	8	3.3	**0.01**
Yes	196	91.2	19	8.8		59	92.2	5	7.8		255	91.4	24	8.6	
**Resident permit at time of survey**															
Undocumented	277	94.5	16	5.5	**0.07**	83	94.3	5	5.7	0.77	360	94.5	21	5.5	**0.07**
Short-term permit (<1 year)	65	95.6	3	4.4		16	94.1	1	5.9		81	95.3	4	4.7	
Long-term permit (1 year and +, including French nationality)	28	84.8	5	15.2		18	90.0	2	10.0		46	86.8	7	13.2	
**Have children**															
No	143	96.6	5	3.4	**0.08**	40	93.0	3	7.0	0.84	183	95.8	8	4.2	**0.15**
Yes	227	92.3	19	7.7		77	93.9	5	6.1		304	92.7	24	7.3	
**Health insurance coverage at time of survey**															

State Medical Assistance (SMA)	97	93.3	7	6.7	0.27	36	92.3	3	7.7	0.66	133	93.0	10	7.0	**0.18**
No Health insurance Coverage	181	95.8	8	4.2		48	96.0	2	4.0		229	95.8	10	4.2	
Universal Health insurance Coverage (UHC)	92	91.1	9	8.9		33	91.7	3	8.3		125	91.2	12	8.8	
**Empowerment scores**															
Low (1st quartile)	84	96.6	3	3.4	**0.02**	35	94.6	2	5.4	0.90	119	96.0	5	4.0	**0.04**
Intermediate low (2nd quartile)	106	98.1	2	1.9		35	94.6	2	5.4		141	97.2	4	2.8	
Intermediate high (3rd quartile)	103	89.6	12	10.4		29	93.5	2	6.5		132	90.4	14	9.6	
High (4th quartile)	77	91.7	7	8.3		18	90.0	2	10.0		95	91.3	9	8.7	
**Have at least one stable partnership**															
No	231	95.5	11	4.5	**0.10**	44	93.6	3	6.4	0.99	275	95.2	14	4.8	**0.16**
Yes	139	91.4	13	8.6		73	93.6	5	6.4		212	92.2	18	7.8	
**Have at least one stable or occasional same-sex sexual partnership in the last 12 months**															
No	263	92.3	22	7.7	**0.09**	99	93.4	7	6.6	0.92	362	92.6	29	7.4	**0.11**
Yes	8	100.0	0	0.0		2	100.0	0	0.0		10	100.0	0	0.0	
No sexual partner	99	98.0	2	2.0		16	94.1	1	5.9		115	97.5	3	2.5	
**Occasional partnership in the last 12 months**															
No	178	93.7	12	6.3	0.85	69	95.8	3	4.2	0.23	247	94.3	15	5.7	0.67
Yes	192	94.1	12	5.9		48	90.6	5	9.4		240	93.4	17	6.6	
**Transactional sex**															
No	357	93.9	23	6.1	0.86	96	96.0	4	4.0	**0.02**	453	94.4	27	5.6	**0.07**
Yes	13	92.9	1	7.1		21	84.0	4	16.0		34	87.2	5	12.8	
**Forced sex**															
No	369	93.9	24	6.1	0.79	110	93.2	8	6.8	0.47	479	93.7	32	6.3	0.46
Yes	1	100.0	0	0.0		7	100.0	0	0.0		8	100.0	0	0.0	

Knowledge of PrEP

	PrEP Men _ (N=394)					PrEP Women _ (N=125)					PrEP All _ (N=519)				
	Non		Oui		p	Non		Oui		p	Non		Oui		p
	N	%	N	%		N	%	N	%		N	%	N	%	
**Sex**															
Men											374	94.9	20	5.1	0.81
Women											118	94.4	7	5.6	
**Age (years)**															
18 – 29	98	96.1	4	3.9	0.40	46	90.2	5	9.8	0.23	144	94.1	9	5.9	0.52
30 – 39	167	93.3	12	6.7		40	97.6	1	2.4		207	94.1	13	5.9	
40 +	109	96.5	4	3.5		32	97.0	1	3.0		141	96.6	5	3.4	
**Educational level**															
None/Primary	116	96.7	4	3.3	0.27	39	97.5	1	2.5	0.49	155	96.9	5	3.1	0.27
Secondary	193	93.2	14	6.8		60	93.8	4	6.3		253	93.4	18	6.6	
Superior	65	97.0	2	3.0		19	90.5	2	9.5		84	95.5	4	4.5	
**Region of birth**															
West Africa	237	94.8	13	5.2	0.88	63	94.0	4	6.0	0.84	300	94.6	17	5.4	0.83
Other part of sub-Saharan Africa	137	95.1	7	4.9		55	94.8	3	5.2		192	95.0	10	5.0	
**Main reason for coming to France**															
Find work/study	179	95.7	8	4.3	0.68	55	93.2	4	6.8	**0.05**	234	95.1	12	4.9	0.41
Join a family member	29	90.6	3	9.4		13	100.0	0	0.0		42	93.3	3	6.7	
Medical reasons and other	16	94.1	1	5.9		6	75.0	2	25.0		22	88.0	3	12.0	
Threatened in your country	150	94.9	8	5.1		44	97.8	1	2.2		194	95.6	9	4.4	
**Duration of stay in France (years)**															
0 – 2	190	96.0	8	4.0	**0.15**	53	96.4	2	3.6	**0.16**	243	96.0	10	4.0	0.39
3 – 6	131	95.6	6	4.4		44	89.8	5	10.2		175	94.1	11	5.9	
7 +	53	89.8	6	10.2		21	100.0	0	0.0		74	92.5	6	7.5	
**Housing situation at time of survey**															
Associations	19	90.5	2	9.5	0.67	24	100.0	0	0.0	0.34	43	95.6	2	4.4	0.66

Housed by family/friends	202	94.4	12	5.6		48	90.6	5	9.4		250	93.6	17	6.4	
Own housing	102	96.2	4	3.8		40	95.2	2	4.8		142	95.9	6	4.1	
No stable housing	51	96.2	2	3.8		6	100.0	0	0.0		57	96.6	2	3.4	
**Occupational status at time of survey**															
Unemployed	248	95.0	13	5.0	0.90	84	94.4	5	5.6	0.98	332	94.9	18	5.1	0.93
Employed (informal/formal/student)	126	94.7	7	5.3		34	94.4	2	5.6		160	94.7	9	5.3	
**Have someone close you can rely on in the times of hardship**															
No	175	97.8	4	2.2	**0.01**	58	95.1	3	4.9	0.74	233	97.1	7	2.9	**0.03**
Yes	199	92.6	16	7.4		60	93.8	4	6.3		259	92.8	20	7.2	
**Resident permit at time of survey**															
Undocumented	279	95.2	14	4.8	0.54	82	93.2	6	6.8	0.53	361	94.8	20	5.2	0.58
Short-term permit (<1 year)	65	95.6	3	4.4		17	100.0	0	0.0		82	96.5	3	3.5	
Long-term permit (1 year and +, including French nationality)	30	90.9	3	9.1		19	95.0	1	5.0		49	92.5	4	7.5	
**Have children**															
No	144	97.3	4	2.7	**0.09**	40	93.0	3	7.0	0.62	184	96.3	7	3.7	0.22
Yes	230	93.5	16	6.5		78	95.1	4	4.9		308	93.9	20	6.1	
**Health insurance coverage at time of survey**															
State Medical Assistance (SMA)	96	92.3	8	7.7	0.36	36	92.3	3	7.7	0.35	132	92.3	11	7.7	0.24
No Health insurance Coverage	181	95.8	8	4.2		49	98.0	1	2.0		230	96.2	9	3.8	
Universal Health insurance Coverage (UHC)	97	96.0	4	4.0		33	91.7	3	8.3		130	94.9	7	5.1	
**Empowerment scores**															
Low (1st quartile)	83	95.4	4	4.6	0.23	36	97.3	1	2.7	0.55	119	96.0	5	4.0	0.46
Intermediate low (2nd quartile)	106	98.1	2	1.9		34	91.9	3	8.1		140	96.6	5	3.4	

Intermediate high (3rd quartile)	106	92.2	9	7.8		30	96.8	1	3.2		136	93.2	10	6.8	
High (4th quartile)	79	94.0	5	6.0		18	90.0	2	10.0		97	93.3	7	6.7	
**Have at least one stable partnership**															
No	234	96.7	8	3.3	**0.04**	45	95.7	2	4.3	0.61	279	96.5	10	3.5	**0.04**
Yes	140	92.1	12	7.9		73	93.6	5	6.4		213	92.6	17	7.4	
**Have at least one stable or occasional same-sex sexual partnership in the last 12 months**															
No	266	93.3	19	6.7	**0.06**	99	93.4	7	6.6	0.51	365	93.4	26	6.6	**0.03**
Yes	8	100.0	0	0.0		2	100.0	0	0.0		10	100.0	0	0.0	
No sexual partner	100	99.0	1	1.0		17	100.0	0	0.0		117	99.2	1	0.8	
**Occasional partnership in the last 12 months**															
No	180	94.7	10	5.3	0.87	69	95.8	3	4.2	0.41	116	95.9	5	4.1	0.52
Yes	194	95.1	10	4.9		49	92.5	4	7.5		128	94.1	8	5.9	
**Transactional sex**															
No	361	95.0	19	5.0	0.72	96	96.0	4	4.0	**0.12**	457	95.2	23	4.8	**0.13**
Yes	13	92.9	1	7.1		22	88.0	3	12.0		35	89.7	4	10.3	
**Forced sex**															
No	373	94.9	20	5.1	0.81	111	94.1	7	5.9	0.50	484	94.7	27	5.3	0.50
Yes	1	100.0	0	0.0		7	100.0	0	0.0		8	100.0	0	0.0	

Source : Makasi survey, 2019-2020
