# Supplementary material for: Low knowledge of antiretroviral treatments for the prevention of HIV among precarious immigrants from sub-Saharan Africa living in the greater Paris area: Results from the Makasi project
Source: PLoS One. 2023 Jun 14;18(6):e0287288. doi: 10.1371/journal.pone.0287288 (PMC10266671; doi:10.1371/journal.pone.0287288)
Supplement: S2 Table — (PDF) [file pone.0287288.s004.pdf]

S 3: Sociodemographic characteristics of those eligible for Makasi, by whether or not they were included in the study (N=1854)

	Not included (N=1253)		Included (N=601)		
	**N**	**%**	**N**	**%**	**p-values**
**Sociodemographic characteristics**					
**Sex**					
Men	834	66.5	458	76.2	<0.001
Women	419	33.4	143	23.8	
**Age (years)**					
18 – 29	409	32.6	184	30.6	<0.001
30 – 39	420	33.5	252	41.9	
40 +	408	32.5	165	27.5	
Missing	16	1.2	-	-	
**Region of birth**					
West Africa	716	57.1	365	60.7	0.223
Other part of sub-Saharan Africa	535	42.7	236	39.3	
Missing	2	0.1	-	-	
**Duration of stay in France (years)**					
0 – 2	386	30.8	301	50.1	<0.001
3 – 6	464	37.0	208	34.6	
7 +	403	32.1	92	15.3	
**Social situation**					
**Housing situation at time of survey**					
Associations	95	7.5	55	9.2	<0.001
Housed by family/friends	485	38.7	303	50.4	
Own housing	599	47.8	167	27.8	
No stable housing	59	4.7	76	12.6	
Missing	15	1.2	-	-	
**Occupational status at time of survey**					
Unemployed	747	59.6	416	69.2	<0.001
Employed (informal/formal/student)	501	39.9	185	30.8	
Missing	5	0.4	-	-	
**Have someone close you can rely on in the times of hardship**					
No	403	32.1	302	50.2	<0.001
Yes	823	65.6	299	49.8	
Missing	27	2.1	-	-	
**Resident permit at time of survey**					
Undocumented	610	48.6	444	73.9	<0.001
Short-term permit (<1 year)	234	18.6	96	16.0	
Long-term permit (1 year and +, including French nationality)	409	32.6	61	10.1	
**Health insurance coverage at time of survey**					
State Medical Assistance (SMA)	268	21.3	162	27.0	<0.001
No Health insurance Coverage	362	28.8	277	46.1	
Universal Health insurance Coverage (UHC)	623	49.7	162	27.0	

Source: Makasi survey, 2019-2020
