# Supplementary material for: Low knowledge of antiretroviral treatments for the prevention of HIV among precarious immigrants from sub-Saharan Africa living in the greater Paris area: Results from the Makasi project
Source: PLoS One. 2023 Jun 14;18(6):e0287288. doi: 10.1371/journal.pone.0287288 (PMC10266671; doi:10.1371/journal.pone.0287288)
Supplement: S3 Table — Nested logistic regression models. (PDF) [file pone.0287288.s005.pdf]

S 4: Factors associated with knowledge of HIV treatment effectiveness. Nested logistic regression models

		**HIV treatment effectiveness** (**N=601)**				
		**bivariate**	**Multivariate**			
			Model 1	Model 2	Model 3	Full model
	**% (n/N)**	OR [95% CI]	aOR [95% CI]	aOR [95% CI]	aOR [95% CI]	aOR [95% CI]
**Sex**						
Men	84.9 (389/458)	1.49++[.92- 2.41]	1.58++[0.94-2.64]	1.79*[1.05-3.04]	1.78*[1.04-3.03]	2.12**[1.22-3.69]
Women	79.0 (113/143)	1.00	1.00	1.00	1.00	1.00
**Age (years)**						
18 – 29	81.5 (150/184)	1.00	1.00	1.00	1.00	1.00
30 – 39	84.5 (213/252)	1.23[.74- 2.05]	1.21[0.70-2.11]	0.91[0.51-1.63]	0.90[0.50-1.62]	0.87[0.48-1.58]
40 +	84.2 (139/165)	1.21[.69- 2.12]	1.06[0.57-1.97]	0.59+[0.28-1.21]	0.57+[0.28-1.18]	0.58+[0.28-1.20]
**Educational level**						
None/Primary	70.9 (129/182)	1.00	1.00		1.00	1.00
Secondary	88.0 (271/308)	3.00***[1.88- 4.81]	2.75***[1.63-4.66]	3.08***[1.79-5.31]	2.99***[1.73-5.15]	2.98***[1.71-5.18]
Superior	91.9 (102/111)	4.65***[2.19- 9.88]	3.89**[1.71-8.88]	4.54***[1.97-10.48]	4.43***[1.92-10.23]	4.33***[1.87-10.04]
**Region of birth**						

West Africa	78.9 (288/365)	1.00	1.00	1.00	1.00	1.00
Other part of sub-Saharan Africa	90.7 (214/236)	2.60***[1.56- 4.31]	1.74++[0.98-3.10]	1.57+[0.87-2.83]	1.53+[0.84-2.76]	1.55+[0.85-2.83]
**Main reason for coming to France**						
Find work/study	82.4 (234/284)	1.20[.56- 2.56]	1.50[0.68-3.34]	1.52[0.67-3.45]	1.67[0.73-3.84]	1.77+[0.76-4.11]
Join a family member	79.6 (39/49)	1.00	1.00	1.00	1.00	1.00
Medical reasons and other	90.0 (27/30)	2.30[.58- 9.17]	1.63[0.38-6.88]	1.90[0.43-8.36]	1.91[0.43-8.44]	1.98[0.45-8.72]
Threatened in your country	84.9 (202/238)	1.43[.65- 3.13]	1.64[0.72-3.73]	1.50[0.64-3.49]	1.64[0.70-3.84]	1.70[0.71-4.03]
**Duration of stay in France (years)**						
0 – 2	82.1 (247/301)	1.00	1.00	1.00	1.00	1.00
3 – 6	84.1 (175/208)	1.15[.72- 1.86]	0.88[0.53-1.47]	0.81[0.47-1.38]	0.77[0.45-1.32]	0.71[0.41-1.22]
7 +	87.0 (80/92)	1.45[.74- 2.86]	1.27[0.61-2.65]	1.17[0.54-2.51]	1.09[0.50-2.36]	1.07[0.49-2.34]
**Have children**						
No	77.6 (170/219)	1.00		1.00	1.00	1.00
Yes	86.9 (332/382)	1.91**[1.23- 2.95]		2.44**[1.43-4.19]	2.43**[1.42-4.17]	2.37**[1.38-4.09]
**Health insurance coverage at time of survey**						
State Medical Assistance (SMA)	87.0 (141/162)	1.77*[1.03- 3.05]		2.01*[1.11-3.63]	1.98*[1.09-3.59]	1.89*[1.04-3.44]

No Health insurance Coverage	79.1 (219/277)	1.00		1.00	1.00	1.00
Universal Health insurance Coverage (UHC)	87.7 (142/162)	1.88*[1.08- 3.26]		2.00*[1.10-3.63]	1.99*[1.09-3.61]	1.93*[1.05-3.53]
**Have someone close you can rely on in the times of hardship**						
No	79.8 (241/302)	1.00			1.00	1.00
Yes	87.3 (261/299)	1.73**[1.11- 2.70]			1.42+[0.87-2.31]	1.28[0.77-2.10]
**Have at least one stable partnership**						
No	79.6 (270/339)	1.00				1.00
Yes	88.5 (232/262)	1.97**[1.24- 3.14]				1.93*[1.14-3.26]
Hosmer–Lemeshow goodness fit test (p-value)			p=0.56	p=0.83	p=0.08	p=0.13
Area under ROC curve			0.6967	0.7304	0.7336	0.7449

Source : Makasi survey, 2019-2020

Model 1: adjusted for all sociodemographic characteristics

Model 2: adjusted for all sociodemographic characteristics and variables related to the contact with the health-care system significant at 20%

Model 3: adjusted for all sociodemographic characteristics and variables related to the contact with the health-care system and the social situation in France significant at 20%.

Full model: adjusted for all sociodemographic characteristics and variables related to the contact with the health-care system, the social situation in France and sexual behaviors significant at 20%.

+ p<0.20, ++ p<0.10, * p<0.05, ** p<0.01, *** p<0.001
