# Supplementary material for: Low knowledge of antiretroviral treatments for the prevention of HIV among precarious immigrants from sub-Saharan Africa living in the greater Paris area: Results from the Makasi project
Source: PLoS One. 2023 Jun 14;18(6):e0287288. doi: 10.1371/journal.pone.0287288 (PMC10266671; doi:10.1371/journal.pone.0287288)
Supplement: S4 Table — Nested logistic regression models. (PDF) [file pone.0287288.s006.pdf]

S 5: Factors associated with knowledge of treatment as prevention (TasP). Nested logistic regression models

		**TasP (N=601)**			
		**Bivariate**	**Multivariate**		
			Model 1	Model 2	Full model
	**% (n/N)**	OR [95% CI]	aOR [95% CI]	aOR [95% CI]	aOR [95% CI]
**Sex**					
Men	46.7 (214/458)	1.21[0.83- 1.77]	1.18[0.80-1.75]	1.04[0.69-1.56]	1.27[0.83-1.95]
Women	41.9 (60/143)	1.00	1.00	1.00	1.00
**Age (years)**					
18 – 29	43.5 (80/184)	1.00	1.00	1.00	1.00
30 – 39	45.2 (114/252)	1.07[.73- 1.57]	1.06[0.71-1.60]	1.05[0.70-1.58]	1.03[0.68-1.56]
40 +	48.5 (80/165)	1.22[.80- 1.86]	1.19[0.75-1.87]	1.18[0.74-1.86]	1.22[0.76-1.95]
**Educational level**					
None/Primary	38.5 (70/182)	1.00	1.00	1.00	1.00
Secondary	48.1(148/308)	1.48*[1.01-2.14]	1.52*[1.01-2.30]	1.51++[0.99-2.30]	1.51++[0.99-2.32]

Superior	50.5(56/111)	1.62*[1.01- 2.62]	1.71*[1.00-2.91]	1.67++[0.97-2.88]	1.66++[0.95-2.89]
**Region of birth**					
West Africa	43.8 (160/365)	1.00	1.00	1.00	1.00
Other part of sub-Saharan Africa	48.3 (114/236)	1.19[.86- 1.66]	1.00[0.69-1.46]	1.00[0.69-1.46]	0.98[0.67-1.43]
**Main reason for coming to France**					
Find work/study	45.1 (128/284)	1.00[.54 – 1.85]	1.08[0.58-2.01]	1.19[0.63-2.24]	1.31[0.69-2.49]
Join a family member	44.9 (22/49)	1.00	1.00	1.00	1.00
Medical reasons and other	43.3 (13/30)	0.93[0.37-2.34]	0.86[0.33-2.22]	0.90[0.34-2.34]	0.98[0.37-2.58]
Threatened in your country	46.6 (111/238)	1.07[0.57-1.98]	1.11[0.59-2.10]	1.19[0.62-2.26]	1.25[0.65-2.39]
**Duration of stay in France (years)**					
0 – 2	45.2 (136/301)	1.00	1.00	1.00	1.00
3 – 6	45.2 (94/208)	1.00[.70- 1.42]	0.92[0.64-1.33]	0.95[0.65-1.39]	0.89[0.60-1.31]
7 +	47.8 (44/92)	1.11[.69- 1.77]	1.04[0.63-1.72]	1.03[0.61-1.72]	1.01[0.60-1.70]

**Housing situation at time of survey**					
Associations	27.3 (15/55)	0.41**[0.21- 0.81]		0.47*[0.23-0.94]	0.41*[0.20-0.82]
Housed by family/friends	46.2 (140/303)	0.95[0.65-1.39]		0.99[0.67-1.47]	1.01[0.68-1.51]
Own housing	47.3 (79/167)	1.00		1.00	1.00
No stable housing	52.6 (40/76)	1.23[0.71-2.13]		1.52+[0.83-2.79]	1.63+[0.88-3.02]
**Have someone close you can rely on in the times of hardship**					
No	42.4 (128/302)	1.00		1.00	1.00
Yes	48.8 (146/299)	1.29+[.94- 1.78]		1.28+[0.90-1.82]	1.19[0.83-1.71]
**Have at least one stable partnership**					
No	40.1 (136/339)	1.00			1.00
Yes	52.7(138/262)	1.66***[1.19- 2.30]			1.90***[1.33-2.73]
**Forced sex**					
No	45.1 (264/585)	1.00			1.00
Yes	62.5 (10/16)	2.02+[0.72- 5.64]			3.17*[1.03-9.69]

Hosmer–Lemeshow goodness fit test (p-value)			p=0.48	p=0.73	p=0.78
Area under ROC curve			0.5622	0.6062	0.6278

Source : Makasi survey, 2019-2020

Model 1: adjusted for all sociodemographic characteristics

Model 2: adjusted for all sociodemographic characteristics and variables related to the social situation in France significant at 20%.

Full model: adjusted for all sociodemographic characteristics and variables related to the social situation in France and sexual behaviors significant at 20%.

+ p<0.20, ++ p<0.10, * p<0.05, ** p<0.01, *** p<0.001
