# Supplementary material for: Low knowledge of antiretroviral treatments for the prevention of HIV among precarious immigrants from sub-Saharan Africa living in the greater Paris area: Results from the Makasi project
Source: PLoS One. 2023 Jun 14;18(6):e0287288. doi: 10.1371/journal.pone.0287288 (PMC10266671; doi:10.1371/journal.pone.0287288)
Supplement: S5 Table — Nested logistic regression models. (PDF) [file pone.0287288.s007.pdf]

S 6: Factors associated with knowledge of post-exposure prophylaxis (PEP). Nested logistic regression models

		**PEP (N=519)**				
		**Bivariate**	**Multivariate**			
			Model 1	Model 2	Model 3	Full model
	**% (n/N)**	OR [95% CI]	aOR [95% CI]	aOR [95% CI]	aOR [95% CI]	aOR [95% CI]
**Sex**						
Men	6.1 (24/394)	0.94[0.41-2.16]	0.88[0.37-2.12]	0.92[0.38-2.23]	0.97[0.38-2.46]	1.64[0.57-4.73]
Women	6.4 (8/125)	1.00	1.00	1.00	1.00	1.00
**Age (years)**						
18 – 29	5.9 (9/153)	1.00	1.00	1.00	1.00	1.00
30 – 39	4.5 (10/220)	0.76[0.30-1.92]	0.88[0.33-2.35]	0.72[0.26-1.99]	0.78[0.26-2.28]	0.79[0.26-2.40]
40 +	8.9 (13/146)	1.56[0.64-3.77]	1.61[0.60-4.30]	1.06[0.36-3.10]	1.04[0.32-3.29]	1.11[0.34-3.61]
**Educational level**						
None/Primary	3.1 (5/160)	1.00	1.00	1.00	1.00	1.00
Secondary	8.9 (24/271)	3.01*[1.12-8.05]	3.85*[1.36-10.90]	4.05**[1.42-11.54]	3.35*[1.08-10.32]	3.33*[1.09-10.20]
Superior	3.4 (3/88)	1.09[.25-4.69]	1.36[0.28-6.57]	1.42[0.29-6.99]	1.17[0.22-6.23]	1.22[0.22-6.57]
**Region of birth**						

West Africa	6.9 (22/317)	1.00	1.00	1.00	1.00	1.00
Other part of sub-Saharan Africa	5.0 (10/202)	0.69[0.32-1.50]	0.46++[0.20-1.07]	0.42++[0.18-1.00]"	0.33*[0.13-0.84]	0.32*[0.12-0.81]
**Main reason for coming to France**						
Find work/study	6.1 (15/246)	0.90[0.25-3.27]	1.16[0.31-4.34]	1.25[0.32-4.83]	1.42[0.32-6.22]	1.50[0.34-6.67]
Join a family member	6.7 (3/45)	1.00	1.00	1.00	1.00	1.00
Medical reasons and other	8.0 (2/25)	1.21[0.18-7.82]	1.79[0.25-12.55]	1.96[0.27-14.18]	1.40[0.16-11.78]	1.41[0.16-12.51]
Threatened in your country	5.9 (12/203)	0.87[0.23-3.25]	1.34[0.33-5.33]	1.33[0.33-5.39]	1.48[0.31-6.95]	1.63[0.34-7.82]
**Duration of stay in France (years)**						
0 – 2	5.1 (13/253)	1.00	1.00	1.00	1.00	1.00
3 – 6	5.4 (10/186)	1.04[.44-2.44]	0.92[0.38-2.20]	0.90[0.36-2.25]	1.02[0.38-2.71]	0.85[0.31-2.34]
7 +	11.3 (9/80)	2.34++[.96-5.69]	2.19+[0.82-5.89]	1.91[0.69-5.27]	2.21+[0.66-7.34]	2.04[0.59-7.00]
**Have children**						
No	4.2 (8/191)	1.00		1.00	1.00	1.00
Yes	7.3 (24/328)	1.80++[.79-4.10]		2.15+[0.82-5.62]	2.04+[0.75-5.49]	2.68++[0.93-7.68]
**Health insurance coverage at time of survey**						
State Medical Assistance (SMA)	7.0 (10/143)	1.72[.69-4.24]		1.67[0.64-4.33]	1.64[0.62-4.37]	1.98+[0.72-5.42]
No Health insurance Coverage	4.2 (10/239)	1.00		1.00	1.00	1.00
Universal Health insurance Coverage (UHC)	8.8 (12/137)	2.19+[.92-5.23]		1.81[0.71-4.62]	0.81[0.23-2.88]	0.74[0.20-2.78]

**Occupational status at time of survey**						
Unemployed	7.7 (27/350)	2.74*[1.03-7.25]			4.70**[1.58-13.96]	4.97**[1.62-15.18]
Employed (informal/formal/student)	3.0 (5/169)	1.00			1.00	réf
**Have someone close you can rely on in the times of hardship**						
No	3.3 (8/240)	1.00			1.00	1.00
Yes	8.6 (24/279)	2.72**[1.20-6.19]			3.07*[1.24-7.60]	3.34*[1.30-8.59]
**Resident permit at time of survey**						
Undocumented	5.5 (21/381)	1.18[.39-3.53]			1.07 [0.30-3.78]	1.01[0.27-3.67]
Short-term permit (<1 year)	4.7 (4/85)	1.00			1.00	1.00
Long-term permit (1 year and +, including French nationality)	13.2 (7/53)	3.08++[.85-11.09]			2.22[.39-12.37]	2.93[.50-17.14]
**Empowerment scores**						
Low (1st quartile)	4.0 (5/124)	1.48[.38-5.64]			2.22[0.53-9.24]	2.45[0.57-10.52]
Intermediate low (2nd quartile)	2.8 (4/145)	1.00			1.00	1.00
Intermediate high (3rd quartile)	9.6 (14/146)	3.73*[1.20-11.64]			4.29*[1.26-14.57]	4.51*[1.30-15.60]
High (4th quartile)	8.7 (9/104)	3.33*[.99-11.15]			2.84+[0.78-10.30]	2.66+[0.72-9.83]
**Have at least one stable partnership**						
No	4.8 (14/289)	1.00				1.00

Yes	7.8 (18/230)	1.66+[.81-3.42]				1.68[0.73-3.84]
**Transactional sex**						
No	5.6 (27/480)	1.00				1.00
Yes	12.8 (5/39)	2.46++[.89-6.81]				5.72**[1.51-21.57]
Hosmer–Lemeshow goodness fit test (p-value)			p=0.64	p=0.23	p=0.41	p=0.29
Area under ROC curve			0.7070	0.7140	0.7882	0.8234

Source : Makasi survey, 2019-2020

Model 1: adjusted for all sociodemographic characteristics

Model 2: adjusted for all sociodemographic characteristics and variables related to the contact with the health-care system significant at 20%

Model 3: adjusted for all sociodemographic characteristics and variables related to the contact with the health-care system and the social situation in France significant at 20%.

Full model: adjusted for all sociodemographic characteristics and variables related to the contact with the health-care system, the social situation in France and sexual behaviors significant at 20%.

+ p<0.20, ++ p<0.10, * p<0.05, ** p<0.01, *** p<0.001
