# Supplementary material for: Low knowledge of antiretroviral treatments for the prevention of HIV among precarious immigrants from sub-Saharan Africa living in the greater Paris area: Results from the Makasi project
Source: PLoS One. 2023 Jun 14;18(6):e0287288. doi: 10.1371/journal.pone.0287288 (PMC10266671; doi:10.1371/journal.pone.0287288)
Supplement: S6 Table — Nested logistic regression models. (PDF) [file pone.0287288.s008.pdf]

S 7: Factors associated with knowledge of pre-exposure prophylaxis (PrEP). Nested logistic regression models

		PrEP (N=519)			
		**Bivariate**	**Multivariate**		
			Model 1	Model 2	Full model
	**% (n/N)**	OR [95% CI]	aOR [95%] CI	aOR [95% CI]	aOR [95% CI]
**Sex**					
Men	5.1 (20/394)	0.90[0.37-2.18]	0.92[0.36-2.29]	0.87[0.35-2.18]	1.24[0.46-3.38]
Women	5.6 (7/125)	1.00	1.00	1.00	1.00
**Age (years)**					
18 – 29	5.9 (9/153)	1.00	1.00	1.00	1.00
30 – 39	5.9 (13/220)	1.00[0.41-2.41]	1.12[0.44-2.85]	1.06[0.41-2.71]	1.10[0.42-2.90]
40 +	3.4 (5/146)	0.56[0.18-1.73]	0.55[0.16-1.85]	0.52[0.15-1.75]	0.57[0.17-1.97]
**Educational level**					
None/Primary	3.1 (5/160)	1.00	1.00	1.00	1.00
Secondary	6.6 (18/271)	2.20++[0.80-6.06]	2.21+[0.74-6.59]	1.98[0.65-5.96]	1.86[0.61-5.64]
Superior	4.5 (4/88)	1.47[0.38-5.64]	1.25[0.28-5.59]	1.13[0.25-5.07]	1.10[0.24-4.97]
**Region of birth**					

West Africa	5.4 (17/317)	1.00	1.00	1.00	1.00
Other part of sub-Saharan Africa	5.0 (10/202)	0.91[0.41-2.04]	0.74[0.31-1.79]	0.72[0.30-1.75]	0.72[0.30-1.73]
**Main reason for coming to France**					
Find work/study	4.9 (12/246)	0.71[0.19-2.65]	0.78[0.20-2.98]	0.97[0.25-3.74]	1.00[0.26-3.90]
Join a family member	6.7 (3/45)	1.00	1.00	1.00	1.00
Medical reasons and other	12.0 (3/25)	1.90[0.35-10.25]	1.95[0.33-11.33]	2.05[0.35-12.02]	2.30[0.38-13.77]
Threatened in your country	4.4 (9/203)	0.64[0.16-2.50]	0.83[0.20-3.36]	0.99[0.24-4.04]	1.00[0.24-4.11]
**Duration of stay in France (years)**					
0 – 2	4.0 (10/253)	1.00	1.00	1.00	1.00
3 – 6	5.9 (11/186)	1.52[0.63-3.67]	1.41[0.57-3.49]	1.30[0.52-3.23]	1.14[0.45-2.90]
7 +	7.5 (6/80)	1.97[0.69-5.60]	2.22+[0.72-6.84]	1.95[0.63-6.03]	1.73[0.55-5.40]
**Have someone close you can rely on in the times of hardship**					
No	2.9 (7/240)	1.00		1.00	1.00
Yes	7.2 (20/279)	2.57*[1.06-6.18]		2.25++[0.89-5.64]	2.28++[0.88-5.90]
**Have at least one stable partnership**					
No	3.5 (10/289)	1.00			1.00
Yes	7.4 (17/230)	2.22*[0.99-4.96]			1.96+[0.84-4.54]
**Transactional sex**					
No	4.8 (23/480)	1.00			1.00

Yes	10.3 (4/39)	2.27+[0.74-6.93]			2.60+[0.72-9.34]
Hosmer–Lemeshow goodness fit test<br><br>(p-value)			p=0.94	p=0.30	p=0.74
Area under ROC curve			0.6484	0.6967	0.7095

Source : Makasi survey, 2019-2020

Model 1: adjusted for all sociodemographic characteristics

Model 2: adjusted for all sociodemographic characteristics and variables related to the social situation in France significant at 20%.

Full model: adjusted for all sociodemographic characteristics and variables related to the social situation in France and sexual behaviors significant at 20%.

+ p<0.20, ++ p<0.10, * p<0.05, ** p<0.01, *** p<0.001
